# Psychological Burden and Quality of Life After Pediatric Liver Transplantation: A Cross-Sectional Study

**DOI:** 10.3390/jcm15113994

**Published:** 2026-05-22

**Authors:** Serkan Suren, Deniz Yavuz Baskiran, Irem Tulum, Adil Baskiran, Sezai Yilmaz

**Affiliations:** 1 Department of Child and Adolescent Psychiatry, Private Clinic, Samsun 55900, Türkiye; drserkansuren@hotmail.com (S.S.); iremttulum@gmail.com (I.T.); 2 Liver Transplantation Institute, Inonu University, Malatya 44280, Türkiye; dr.adil.baskiran@gmail.com (A.B.); sezai.yilmaz@inonu.edu.tr (S.Y.)

**Keywords:** pediatric liver transplantation, quality of life, anxiety disorders, post-traumatic stress, child mental health

## Abstract

**Background/Objectives:** Survival rates after pediatric liver transplantation have improved substantially over recent decades, yet the psychiatric consequences for recipients remain a concern that warrants closer attention. We sought to map the psychiatric symptom burden across multiple domains in this population and to determine which symptom clusters carry the greatest impact on health-related quality of life (HRQOL). **Materials and Methods:** Fifty liver transplant recipients between the ages of 8 and 18 were enrolled at a single center. Children and their parents completed four psychiatric measures—the CBCL, CDI, SCARED, and CRIES-13—alongside the parent-proxy PedsQL to capture HRQOL across physical, emotional, social, and school functioning domains. Correlations between instruments were calculated, and linear regression was used to determine which psychiatric variables independently predicted PedsQL Total scores. **Results:** Across all psychiatric measures, higher symptom scores were associated with lower HRQOL, with school functioning recording the lowest absolute PedsQL domain score, while emotional functioning demonstrated the strongest and most consistent inverse correlations with all psychiatric symptom measures across instruments. CBCL Total (r = −0.607), SCARED Total (r = −0.557), and CRIES-13 Total (r = −0.548) scores all correlated meaningfully with overall HRQOL. When entered into multivariable analysis, anxiety symptoms measured by the SCARED (β = −0.295, *p* = 0.032) and post-traumatic stress symptoms measured by the CRIES-13 (β = −0.400, *p* = 0.004) stood out as the two independent predictors of worse PedsQL Total scores. **Conclusions:** Even in medically stable recipients, anxiety and post-traumatic stress symptoms were independently associated with lower daily functioning scores and overall quality of life. These findings suggest that routine psychosocial screening and trauma-informed approaches may warrant integration into post-transplant care protocols, and that prospective, adequately powered studies are needed to confirm and extend these associations.

## 1. Introduction

Pediatric liver transplantation (PLT) has gradually become the standard treatment for children with end-stage liver disease, biliary atresia, and certain metabolic disorders. Data from recent studies indicate that PLT achieves high rates of therapeutic success and has low risks, with 5-year patient survival rates surpassing 85–90% and 20-year survival rates nearing 80% [1,2].

The psychosocial burden of PLT, however, is still a lesser-described aspect. Children and parents endure extended hospitalizations, recurrent invasive procedures, intensive care experiences, lifelong immunosuppression, and persistent anxiety regarding rejection or complications [3,4]. These stressors correlate with increased prevalence of psychiatric symptoms relative to healthy children, including elevated levels of depression (13–31%), anxiety (31–47%), posttraumatic stress disorder (PTSD) symptoms (44–48%), and behavioral challenges [3,5,6]. Parents also report high prevalence of internalizing issues, such as anxiety, depression, and withdrawal [7].

Health-related quality of life (HRQOL) is an essential outcome domain after PLT. Recipients of PLT exhibit significantly poorer quality of life –much like children with other chronic diseases, particularly in areas of school functioning, physical health, and overall health perceptions [8]. HRQOL is negatively associated with psychiatric symptom burden. In cross-sectional studies, elevated scores in depression, anxiety, and PTSD have been consistently observed alongside diminished physical, emotional, social, and academic functioning, though the directionality of these associations remains to be established through longitudinal investigation [3,9]. Clinical factors, including time elapsed since transplantation, frequency of rejection episodes, hospitalization rates, and immunosuppressive burden, further influence both psychiatric symptoms and HRQOL [10].

The majority of current studies utilize parent-report screening tools, such as the Child Behavior Checklist (CBCL) and the Strengths and Difficulties Questionnaire. While useful, these tools lack Diagnostic and Statistical Manual of Mental Disorders (DSM)–level diagnostic accuracy. The Kiddie Schedule for Affective Disorders and Schizophrenia–Present and Lifetime Version and other semi-structured diagnostic interviews have not been used much in the transplant population, even though they can make accurate clinical diagnoses [11,12]. Based on the limitations of available data, there is a need to expand studies in this field by utilizing multiple tools to better understand the psychological impact of PLT. Comprehensive analysis of post-PLT psychiatric burden benefits from the integration of (i) structured diagnostic screening interviews capable of generating DSM-referenced clinical impressions, (ii) dimensional symptom severity measures across multiple domains, (iii) parent-proxy HRQOL assessment, and (iv) analysis of correlations with key clinical parameters [13].

The current cross-sectional study including PLT recipients was designed to examine the prevalence of psychiatric disorders (via K-SADS-PL), the severity of psychiatric symptoms, HRQOL, and the relationships between these variables and multiple clinical factors (time since transplantation, rejection episodes, hospitalization history). Our purpose was to address gaps in the literature by combining diagnostic interviews, multi-domain symptom assessments, and disease-specific HRQOL measurements.

## 2. Materials and Methods

### 2.1. Ethics, Design and Setting

Ethical approval was obtained from the Scientific Research and Publication Ethics Committee of Inonu University (Approval date: 2 December 2025, decision no: 2025/8901) based on the ethical standards described in the Declaration of Helsinki. Before enrolling, all of the children and their parents (or legal guardians) had to sign a written form to provide their consent. It was clearly indicated that participation was voluntary and that participants could leave the study and request data deletion at any time. To safeguard participant confidentiality, all personal identifiers were anonymized during data collection, with analysis and reporting performed on unidentifiable data.

This study utilized a single-center, cross-sectional design to assess psychiatric symptoms, clinical diagnoses, and HRQOL in pediatric liver transplant recipients. The research was conducted at the Liver Transplantation Institute, Inonu University, in Malatya, Türkiye. Data collection took place from December 2025 to January 2026 either during regular outpatient follow-up visits or through structured online interviews when in-person attendance was impractical.

### 2.2. Participants

The study population was planned to include children aged 8 to 16 years who had undergone PLT and attended ongoing follow-up at the participating institution. Participants were recruited through consecutive sampling from the outpatient clinic registry. Of all potentially eligible patients identified during the recruitment period 52 were approached; two declined participation or did not meet inclusion criteria, yielding a final enrolled sample of 50. The criteria for inclusion were: (1) being between the ages of 8 and 18 at the time of the assessment; (2) having had at least one successful liver transplant and being medically stable as confirmed by the treating physician; (3) being able to communicate in Turkish or Arabic; (4) having provided written informed consent; and (5) being willing to complete the required assessments either in person or online. Exclusion criteria were: (1) severe intellectual disability (intelligence quotient < 70, as established by prior clinical records or screening); (2) substantial neurological impairment that would prevent participation in interviews or self-report measures; (3) sensory deficits preventing the completion of assessments; and (4) acute medical instability or hospitalization at the time of recruitment. Fifty children met the requirements and were included.

No formal a priori power calculation was performed, as this study was designed as an exploratory, cross-sectional investigation without a predefined primary outcome or comparison group. The enrolled sample of 50 is consistent with sample sizes reported in comparable single-center psychiatric studies in pediatric liver transplant populations, which have typically ranged from 30 to 62 participants [3,5,7]. To contextualize the adequacy of the sample in response to concerns about statistical stability, post hoc power estimation was performed for the primary correlation analyses. Using the observed effect size (r ≈ −0.55) as a reference, a sample of 50 participants yields approximately 97% power at α = 0.05 (two-tailed), suggesting that the enrolled sample was sufficient to detect associations of the magnitude observed. This estimate should be interpreted descriptively rather than inferentially. Given the exploratory design, all findings should be considered hypothesis generating and subjected to confirmation in adequately powered prospective studies.

### 2.3. Interviews and Data Collection

Data were gathered via structured clinical interviews, self-report questionnaires, parent-proxy reports, and medical record reviews. All tests were administered by specialist trained research staff, such as a child psychiatrist and a clinical psychologist, in a quiet, private space to reduce stress and improve accuracy. For in-person sessions, participants were evaluated during scheduled clinic visits, while online assessments were conducted through secure video conferencing platforms that adhered to data protection regulations. The process started with an explanation of the study’s goals and methods. After recording of sociodemographic and clinical data, psychiatric and quality-of-life assessments were performed. Each session lasted about 60 to 90 min, with breaks as needed, especially for younger kids. To ensure consistency, the contents of the items were kept the same and scale instructions were read aloud with each party confirming accurate comprehension. Positive screening results from initial tools were further assessed via structured diagnostic approaches and all data were entered into a secure electronic database immediately after collection to reduce transcription errors. Although standardized administration procedures were maintained across both modalities—including scripted instructions, identical item content, and confirmation of comprehension prior to each section—the possibility that online and in-person settings differ in ways that affect psychological self-disclosure or response patterns cannot be fully excluded.

### 2.4. Demographic and Clinical Information

A custom-made form for sociodemographic and medical information was used to gather important data. This included age, sex, height, weight (from which body mass index was calculated), presence of comorbidities, immigrant status, school attendance, parental ages and education levels, family history of chronic diseases, age at transplantation, time since transplantation, donor type (living or deceased), immunosuppressive regimen (either tacrolimus or everolimus), history of transplantation complications, rejection episodes in the prior year, hospitalizations in the prior year, and liver function tests (aspartate aminotransferase [AST], alanine aminotransferase [ALT], total bilirubin, and gamma-glutamyl transferase [GGT]). Data were obtained from electronic medical records and corroborated parents during the interview.

### 2.5. Instruments for Psychiatric Evaluation

Psychiatric evaluations included both diagnostic interviews and scales that focused on specific properties.

The Children’s Depression Inventory (CDI) [14] was used to measure the severity of symptoms more precisely. This 27-item self-report scale questions depressive symptoms over the past two weeks and yields scores from 0 to 54 points, with higher scores meaning more severe symptoms. The cutoffs were: <15 normal, 15–19 mild, 20–24 moderate, and ≥25 severe. The Screen for Child Anxiety Related Emotional Disorders (SCARED) [15] was filled out by both the child and the parent. It had 41 items across five subscales: panic/somatic, generalized anxiety, separation anxiety, social anxiety, and school avoidance. A total score of 25 or higher indicated clinical anxiety, and there were specific thresholds for each subscale to help with interpretation. The Children’s Revised Impact of Event Scale-13 (CRIES-13) [16] assessed PTSD symptoms associated with transplant experiences, comprising 13 items categorized into intrusion, avoidance, and arousal subscales (total scores ≥17 suggesting possible PTSD). Finally, the Child Behavior Checklist (CBCL/6-18) [17], a 113-item parent-report tool, was used to measure a wide range of behavioral and emotional issues. It yielded total, internalizing, and externalizing scores, with T-scores of 65 or higher being clinically significant.

Structured diagnostic screening was conducted using the Kiddie Schedule for Affective Disorders and Schizophrenia—Present and Lifetime Version (K-SADS-PL), administered by a trained child psychiatrist. The K-SADS-PL was administered by a single child psychiatrist (S.S.) with formal training in structured diagnostic interviewing. Both parent and child versions were completed for each participant, in accordance with standard protocol. Interview duration ranged from 45 to 75 min per dyad. No formal inter-rater reliability assessment was performed within this study, which represents a methodological limitation.

### 2.6. Quality of Life

The Pediatric Quality of Life Inventory (PedsQL) 4.0 Generic Core Scales [18], parent-proxy version, was used to measure HRQOL. It has 23 items that cover physical, emotional, social, and school functioning areas. The scores for each subscale range from 0 to 100, with higher scores meaning better quality of life. The total score and psychosocial health summary score are found by averaging the scores for the relevant subscales. Parents filled out the form on their own based on how they thought their child had been doing over the past month. The scales employed in this study are based on Turkish reliability and validity studies. For the seven participants who were Arabic-speaking (14% of the sample), all instruments were administered with the assistance of a trained medical interpreter fluent in both Turkish and Arabic; instructions were read aloud and comprehension was confirmed prior to proceeding.

### 2.7. Statistical Analysis

We used IBM SPSS version 27 (IBM Corp., Armonk, NY, USA) for statistical analyses. Statistical significance was defined as two-tailed *p*-values below 0.05. We used histograms and Q-Q plots to see if continuous variables were normally distributed. Mean ± standard deviation was used to describe normally distributed continuous variables, median (25th percentile–75th percentile) was used to describe non-normally distributed continuous variables, and frequency (percentage) was used for categorical variables. The Pearson or Spearman correlation coefficients were calculated as appropriate to determine effect sizes. We used linear regression analyses to identify clinical and psychiatric variables independently associated with PedsQL Total scores. All variables with clinical plausibility or a univariable *p*-value below 0.10 were eligible for inclusion in the multivariable model. A stepwise selection procedure (entry threshold *p* < 0.05, removal threshold *p* > 0.10) was applied to construct a parsimonious final model. Variance inflation factor (VIF) values were calculated to evaluate multicollinearity; a VIF below 5 was considered acceptable. Prior to regression, key assumptions were assessed: residual normality was examined using P-P plots and the Shapiro–Wilk test, homoscedasticity was evaluated via scatter plots of standardized residuals against predicted values, and the absence of influential outliers was confirmed using Cook’s distance. Both unstandardized (B) and standardized (β) regression coefficients are reported alongside 95% confidence intervals and *p*-values. No correction for multiple comparisons was applied. Findings with *p*-values between 0.025 and 0.050 should be interpreted with particular caution as exploratory signals requiring prospective replication.

## 3. Results

The study comprised 50 participants (22 females) who underwent PLT, with a median age of 14 years (*n* = 11–16). Demographic and clinical characteristics including family data are summarized in Table 1. The most common indication for liver transplantation was acute fulminant hepatic failure, accounting for 21 patients (42.0%), of whom 10 had cryptogenic etiology, seven had decompensated Wilson’s disease, three had hepatitis A-related failure, and two had toxic or toxin-induced failure. Non-fulminant diagnoses included cryptogenic cirrhosis (*n* = 5, 10.0%), non-specific cholestasis (*n* = 5, 10.0%), non-fulminant Wilson’s disease (*n* = 4, 8.0%), Niemann-Pick disease (*n* = 3, 6.0%), and autoimmune hepatitis (*n* = 3, 6.0%). The remaining nine patients (18.0%) carried rarer diagnoses, including progressive familial intrahepatic cholestasis *(n* = 2), biliary atresia (*n* = 2), primary sclerosing cholangitis (*n* = 1), Crigler-Najar syndrome (*n* = 1), congenital hepatic fibrosis (*n* = 1), and other metabolic conditions (*n* = 2).

Psychiatric measures and scale results, including CBCL, CDI, SCARED subscales, CRIES-13, and PedsQL, are described in Table 2. Although dimensional symptom scales identified elevated scores across multiple psychiatric domains, structured diagnostic interview (K-SADS-PL) did not yield formal DSM-based diagnoses in any participant. This likely reflects the higher diagnostic threshold required for a clinical diagnosis relative to the lower cutoffs used for dimensional screening scales, as well as the medically stable nature of the cohort. These findings are consistent with prior reports demonstrating that sub-threshold symptom burden—particularly in anxiety and post-traumatic stress—can meaningfully impair functioning even in the absence of a diagnosable disorder. Applying predefined clinical thresholds, 10 participants (20.0%) had CBCL Total T-scores at or above 65, the cutoff indicative of clinical significance. On the CRIES-13, 17 participants (34.0%) scored 17 or above, the threshold suggestive of possible PTSD. 

The tests in Table 3 involve correlated outcomes across overlapping psychiatric constructs measured in the same participants; standard correction procedures such as Bonferroni assume test independence and would therefore be overly conservative in this context. 

**Figure 1:**
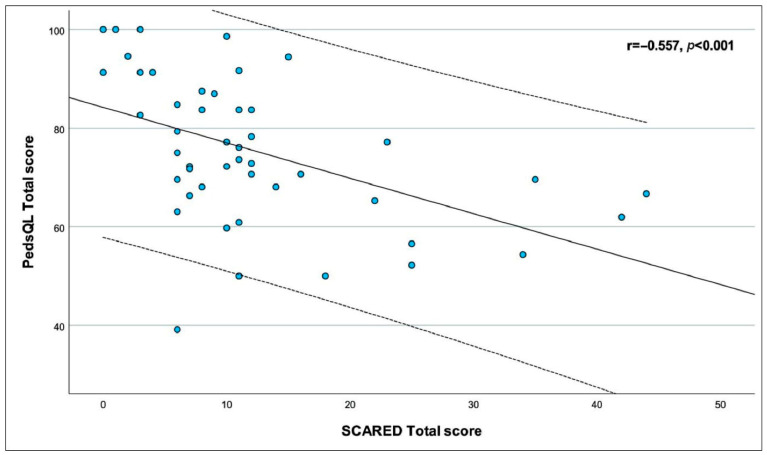
Scatter plot of Pediatric Quality of Life Inventory Total score and Screen for Child Anxiety Related Disorders Total score. Scatter plot of Pediatric Quality of Life Inventory (PedsQL) Total score and Screen for Child Anxiety Related Disorders (SCARED) Total score. Each blue dot represents an individual participant (*n* = 50). The solid line represents the linear fit, and the dotted lines indicate the 95% confidence interval band.

**Figure 2:**
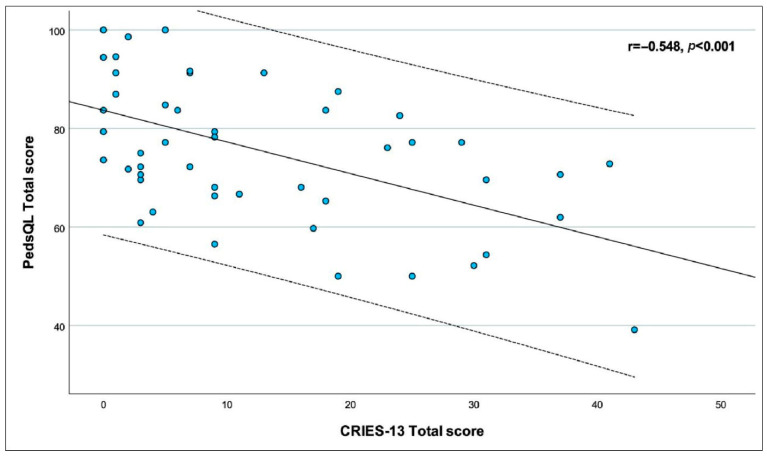
Scatter plot of Pediatric Quality of Life Inventory Total score and Children’s Revised Impact of Events Scale 13-item Total score. Scatter plot of Pediatric Quality of Life Inventory (PedsQL) Total score and Children’s Revised Impact of Events Scale 13-item (CRIES-13) Total score. Each blue dot represents an individual participant (*n* = 50). The solid line represents the linear fit, and the dotted lines indicate the 95% confidence interval band.

Among the PedsQL domains, school functioning recorded the lowest mean score (63.29 ± 19.25), followed by physical health (median 71.88). Emotional functioning yielded the highest median score (92.5, IQR 85–100), reflecting relatively preserved but highly variable emotional ratings. Despite its higher median value, emotional functioning demonstrated the strongest and most consistent inverse correlations with all psychiatric symptom measures, as detailed below. There were strong negative correlations between CBCL Total scores and all PedsQL domains. These included Physical Health (*p* = 0.003, r = −0.417), Emotional Functioning (*p* < 0.001, r = −0.778), Social Functioning (*p* = 0.003, r = −0.406), Psychosocial Health (*p* < 0.001, r = −0.608), and Total PedsQL scores (*p* < 0.001, r = −0.607). Likewise, CDI scores exhibited significant negative correlations with Physical Health (*p* = 0.009, r = −0.365), Emotional Functioning (*p* < 0.001, r = −0.688), Social Functioning (*p* = 0.045, r = −0.284), Psychosocial Health (*p* = 0.001, r = −0.467), and Total PedsQL scores (*p* = 0.001, r = −0.446). The SCARED subscale and total scores exhibited a significant negative correlation with various PedsQL domains. Panic and somatic symptoms were linked to lower scores on the Emotional Functioning (*p* < 0.001, r = −0.678), Psychosocial Health (*p* = 0.005, r = −0.390), and Total PedsQL (*p* = 0.004, r = −0.397) scales. Generalized Anxiety exhibited significant negative correlations with Emotional Functioning (*p* < 0.001, r = −0.757), Psychosocial Health (*p* < 0.001, r = −0.539), and Total scores (*p* = 0.001, r = −0.439). Separation Anxiety exhibited a negative correlation with Emotional Functioning (*p* < 0.001, r = −0.514), Psychosocial Health (*p* = 0.008, r = −0.370), and Total PedsQL scores (*p* = 0.025, r = −0.318). Social Anxiety correlated with diminished Physical Health (*p* < 0.001, r = −0.511), Emotional Functioning (*p* < 0.001, r = −0.516), Social Functioning (*p* = 0.032, r = −0.304), Psychosocial Health (*p* < 0.001, r = −0.480), and Total scores (*p* < 0.001, r = −0.582). School Avoidance had a strong link to Physical Health (*p* = 0.025, r = −0.373), Emotional Functioning (*p* < 0.001, r = −0.595), Psychosocial Health (*p* = 0.013, r = −0.409), and Total PedsQL scores (*p* = 0.005, r = −0.457). The overall SCARED Total scores exhibited substantial negative correlations with Physical Health (*p* = 0.004, r = −0.403), Emotional Functioning (*p* < 0.001, r = −0.745), Social Functioning (*p* = 0.028, r = −0.312), Psychosocial Health (*p* < 0.001, r = −0.510), and Total scores (*p* < 0.001, r = −0.557) (Figure 1). Post-traumatic stress symptoms evaluated by CRIES-13 exhibited significant negative correlations with multiple PedsQL domains. Intrusion scores were significantly correlated with diminished Emotional Functioning (*p* < 0.001, r = −0.642), Social Functioning (*p* = 0.023, r = −0.320), Psychosocial Health (*p* < 0.001, r = −0.522), and Total PedsQL scores (*p* = 0.004, r = −0.400). There was a negative correlation between avoidance scores and Emotional Functioning (*p* < 0.001, r = −0.436), Social Functioning (*p* = 0.007, r = −0.376), Psychosocial Health (*p* = 0.001, r = −0.470), and Total scores (*p* = 0.004, r = −0.396). Arousal exhibited significant negative correlations with Physical Health (*p* = 0.002, r = −0.434), Emotional Functioning (*p* < 0.001, r = −0.665), Psychosocial Health (*p* < 0.001, r = −0.544), and Total PedsQL scores (*p* < 0.001, r = −0.575). In the same way, CRIES-13 Total scores were significantly negatively correlated with Physical Health (*p* = 0.017, r = −0.336), Emotional Functioning (*p* < 0.001, r = −0.676), Social Functioning (*p* = 0.016, r = −0.340), Psychosocial Health (*p* < 0.001, r = −0.605), and Total PedsQL scores (*p* < 0.001, r = −0.548) (Figure 2) (Table 3).

The results of multivariable linear regression analysis showed that a high SCARED Total score (b: −0.431, 95% CI: −0.824–−0.038, *p* = 0.032) and a high CRIES-13 Total score (b: −0.471, 95% CI: −0.787–−0.154, *p* = 0.004) were both linked to lower PedsQL Total scores. The VIF was determined to be 1.321, indicating the absence of multicollinearity in the final model (Table 4).

## 4. Discussion

The present study comprehensively evaluated the psychiatric symptoms experienced by PLT recipients, including emotional and behavioral challenges, anxiety, depression, and post-traumatic stress, which are negatively correlated with multiple aspects of HRQOL. Multiple correlations were found between examined parameters, with findings showing that behavioral issues were correlated with diminished physical health, emotional functioning, social functioning, and overall psychosocial state. Depressive symptoms were associated with worse physical and mental health, while anxiety subscales—particularly social and separation anxiety—were associated with worse emotional and psychosocial outcomes. PTSD characteristics, including intrusion, avoidance, and arousal, showed consistent negative correlations with emotional, social, and overall quality of life scores. Notably, none of the participants had experienced a rejection episode in the preceding year, and only two had required hospitalization, confirming the medically stable nature of this cohort and suggesting that the observed psychiatric burden cannot be attributed to acute graft-related stressors.

PLT is associated with increased rates of psychiatric symptoms and poorer HRQOL, and this inverse relationship is corroborated by the negative correlations between behavioral issues and various quality of life domains [3,6]. For example, Ünay and colleagues found in 50 PLT recipients that 30% to 60% had psychiatric diagnoses. Anxiety and depression symptoms were negatively correlated with PedsQL especially in the emotional and social functioning domains [3]. In a similar vein, Huang et al.’s propensity score-matched analysis of 741 children following living donor liver transplantation revealed heightened emotional issues and hyperactivity, which were associated with diminished psychosocial health, as assessed by the Strengths and Difficulties Questionnaire [6]. In our cohort, the total scores from the CBCL showed strong negative links to physical health (r = −0.417), emotional functioning (r = −0.778), and overall quality of life (r = −0.607), demonstrating that findings in this regard are confirmed by multiple studies.

Chronic immunosuppression may exert neurotoxic effects that disrupt neurotransmitter pathways, including serotonin and dopamine, which are fundamental to mood regulation and behavioral control [9]. Furthermore, pre-PLT nutritional deficiencies and the duration of the disease may impact neurodevelopment in children, leading to cognitive and emotional disruption [19]. Our results show that such impacts remain relevant in a cohort comprised of patients who had undergone successful PLT, suggesting that psychiatric symptoms are associated with diminished quality of life even after successful treatment. It appears that the CBCL may be useful to identify at-risk children, thereby potentially alleviating long-term psychosocial deterioration.

We incorporated a structured screening interview (K-SADS-PL) to systematically assess DSM-referenced psychiatric presentations alongside dimensional symptom scales [20]. Dimensional symptom assessment revealed that internalizing symptoms were particularly elevated in this cohort, even in the absence of formal diagnostic thresholds being met. It can be hypothesized that these outcomes may reflect the cumulative burden of lifelong medical management, including recurrent hospitalizations and immunosuppression. The cumulative burden of recurrent hospitalizations, invasive procedures, and lifelong immunosuppression represents a sustained psychosocial stressor that may perpetuate internalizing symptoms well beyond the acute transplant period, a pattern consistent with observations in other pediatric chronic illness populations [3,7] Although age at transplantation did not emerge as a statistically significant predictor in our multivariable model, the prior literature suggests that earlier transplant age may be associated with greater emotional vulnerability in later childhood. By quantifying these associations in adolescents, it may be possible to design interventions for domain-specific burdens, such as diminished academic performance that has been described in the long term among PLT recipients [21]. Indeed, cognitive-behavioral therapy and family support have been recommended to alleviate adverse psychiatric outcomes [22].

A finding that warrants explicit discussion is the apparent dissociation between school functioning scores and psychiatric symptom burden. School functioning recorded the lowest mean domain score in this cohort (63.29 ± 19.25), yet none of the four psychiatric instruments yielded a statistically significant correlation with this domain, and no psychiatric variable was retained as a predictor of school functioning in the regression analyses. This pattern suggests that school-related impairment in PLT recipients is likely driven by mechanisms outside the psychiatric domain captured by the instruments used here. Neurocognitive sequelae of early-life liver disease—including deficits in processing speed, attention, and working memory—have been well documented in long-term PLT recipients and may impair academic performance independently of mood or anxiety [19]. Structural barriers, including school absence attributable to medical appointments, post-transplant recovery, and ongoing follow-up demands, represent additional contributors that self-report symptom scales are not designed to measure. Post-transplant fatigue and physical deconditioning, which can result from prolonged illness, poor nutritional status, and the effects of ongoing immunosuppression, may also affect school attendance and academic performance through pathways that are independent of mood or anxiety and are not measured by the psychiatric tools used in this study. Of note, 14 of the 50 participants in this cohort (28%) were not attending school at the time of assessment—a proportion that itself reflects the educational disruption associated with chronic illness management and that may have influenced domain scores in ways not captured by the psychiatric instruments. Future studies should incorporate objective neurocognitive assessments and verified school attendance records alongside psychiatric measures to fully characterize the multidetermined nature of academic outcomes in this population.

Anxiety and PTSD were identified as significantly harmful to emotional and psychosocial well-being in our sample, with various subscales demonstrating strong negative correlations with HRQOL domains, corroborating prior evidence from non-transplant populations [23]. Düken and Yayan identified elevated anxiety and post-traumatic stress in young PLT recipients and showed that they correlated with diminished HRQOL, as measured by the PedsQL [5]. Similarly, a single-center study involving 62 PLT recipients found that 9.67% had attention-deficit/hyperactivity disorder through clinical examination, with comorbid anxiety associated with inferior neurocognitive outcomes [7]. Our findings from the SCARED subscales and the total score from the CRIES-13 reveal the influence of anxiety on daily functioning –which are validated by studies involving children with medically-induced trauma (such as intensive care experience) [16].

Social isolation during recovery, as reported in a study of 84 adults who had received liver transplantation in childhood—a population that differs from our pediatric cohort in developmental stage but nevertheless provides relevant long-term outcome data—increases the risk and extent of adverse outcomes. It was found that 26% of these patients experienced mental health disorders associated with early transplant age [22]. We find that anxiety was associated with not only emotional functioning but also social integration. In a prospective cohort study conducted by Mohammad et al., the cessation of immunosuppression was linked to enhancements in disease-specific HRQOL. Notably, this improvement was mitigated in patients with persistence of anxiety symptoms [9]. In comparison, the elevated separation anxiety scores in our cohort may indicate the distinct stressors associated with parental dependency in younger transplant recipients. It must be acknowledged that the regression models presented here did not incorporate several variables with plausible influence on both psychiatric symptom burden and quality of life, including family functioning, socioeconomic circumstances, parental mental health status, and medication adherence. Each of these factors has been independently associated with child psychological outcomes in chronic illness populations. Their absence from the final models means that the predictive associations reported for SCARED and CRIES-13 scores may partly reflect the influence of these unmeasured constructs, and no causal inference is warranted on the basis of this cross-sectional analysis alone. Indeed, Marshall and colleagues showed in adolescents that age at transplantation affected anxiety levels and HRQOL [24]. By integrating the SCARED score in our study [15], we demonstrate that somatic and panic symptoms further diminish physical health [9]. It is also important to note that cultural factors such as family-centric caregiving may increase separation anxiety, as suggested in adolescents [4], which is a crucial factor to consider with regard to the cultural family-centric characteristic of Turkish society. It may be feasible to recommend personalized treatments for such trauma, especially in the presence of comorbidities [7].

Depression and its correlation with impaired physical and emotional health in our cohort appear to be similar to those observed in chronic illness, where mood disorders and family dynamics can impact medical compliance. Among Turkish PLT recipients, depressive symptoms appear to persist even three years post-transplantation and our findings do not question this extensive impact [5]. These long-term influences are also observed in other populations, with younger age at transplantation increasing depressive symptoms later in life, as well as adversely affecting PedsQL domains such as appearance and social functioning [24]. Our findings, which indicate a negative correlation between depressive symptoms and physical and emotional health, are consistent with these observations. Notably, immunosuppressive agents can interfere with serotonin signaling and increase neuroinflammatory processes associated with depression [9]. Furthermore, parental mental health burdens can also cause increased risks for child depression [20]. In our sample, this is demonstrated by the correlations between depressive scores and family environment factors. Indeed, Huang et al. have shown that parental well-being is a predictor of child emotional health [6]. Our comprehensive evaluation reveals that depression is associated with impairment across both emotional and physical health, adding to prior research examining treatment non-adherent individuals [25]. We also show that even stable PLT recipients who adhere to treatments encounter these risks, which may appear to be in conflict to studies examining more acute states [19].

The absence of formal DSM-based diagnoses on structured interview despite elevated dimensional symptom scores warrants careful conceptual interpretation rather than being viewed as a contradictory finding. Contemporary models of child psychopathology increasingly conceptualize psychiatric symptoms as existing along dimensional continua rather than as strictly categorical entities, recognizing that clinically meaningful distress and impairment may emerge well below formal diagnostic thresholds [26]. Instruments such as the SCARED and CRIES-13 are intentionally designed as sensitive screening measures capable of detecting early or sub-threshold internalizing symptoms, whereas the K-SADS-PL requires the full constellation of DSM criteria—including minimum symptom count, duration, pervasiveness, and clinically significant functional impairment—before a diagnosis can be assigned. In medically stable pediatric transplant populations, this distinction may be particularly important. Children may continue to experience persistent anxiety, hypervigilance, illness-related worry, and trauma-associated emotional distress linked to years of medical treatment without necessarily demonstrating the level of symptom persistence or cross-setting impairment required for a formal psychiatric disorder. Similar patterns of elevated sub-diagnostic internalizing burden have been described in other pediatric chronic illness populations, where dimensional symptom elevations are associated with reduced functioning and quality of life despite relatively low rates of categorical diagnosis [3,8,20]. The present findings therefore suggest that reliance on DSM-threshold diagnoses alone may underestimate the psychosocial burden experienced by pediatric liver transplant recipients. From a clinical standpoint, these results argue for a surveillance-based psychosocial model in post-transplant care rather than a diagnosis-triggered referral model alone. Even sub-threshold anxiety and post-traumatic stress symptoms may carry meaningful functional consequences and may justify early supportive intervention before progression to full syndromal disorder.

A methodological consideration warranting brief discussion concerns the heterogeneity of assessment conditions in this study. Forty percent of participants completed assessments via secure video-conferencing, and seven participants required interpreter assistance. In pediatric psychological assessment, the physical and relational context of the evaluation may influence willingness to disclose emotionally sensitive experiences; the reduced immediacy of online settings may lead some children to underreport internalizing symptoms, while others may perceive greater privacy and report more freely. Similarly, although interpreter-mediated administration preserved item content and comprehension was confirmed before each section, the interpersonal dynamics of trilateral communication—particularly for emotionally loaded items concerning anxiety and post-traumatic stress—differ from direct clinician–patient interaction. These contextual differences may have introduced systematic variation in responses that the regression model was not powered to detect.

### Limitations

Several limitations of this study warrant consideration. The cross-sectional design is the most fundamental constraint, as it precludes any causal interpretation of the associations observed between psychiatric symptoms and HRQOL; the direction of these relationships cannot be established from a single time-point assessment. Relatedly, participants were recruited from those actively attending routine outpatient follow-up and were required to be medically stable at the time of assessment. This constitutes a deliberate but consequential selection constraint: children with more severe clinical or psychological conditions—including those lost to follow-up, acutely unwell, non-adherent to monitoring, or unable to attend clinic—are systematically excluded. The psychiatric burden reported here therefore likely represents a conservative estimate of what exists across the full PLT population. Registry-based or multi-center studies that capture patients regardless of follow-up attendance are needed to determine the true population-level psychiatric burden in this group. The single-center design further limits generalizability, and replication across multiple centers and more diverse populations is needed. More than sixty pairwise correlation tests were conducted in Table 3 without correction for multiple comparisons. While correction procedures are not straightforwardly applicable to a matrix of correlated outcomes in an exploratory design, borderline-significant associations (*p* = 0.025–0.050) should be treated as hypothesis-generating signals pending replication in independent samples.

Reliance on self-report and parent-proxy measures introduces the possibility of systematic reporting bias; adolescents may underreport symptoms to avoid stigma, while parents may overestimate difficulties out of concern. Although interview modality was not a statistically significant predictor of HRQOL in univariable analysis, we cannot exclude the possibility that online participation introduced subtle differences in response patterns for emotionally sensitive items that were not captured by the regression model. For the seven Arabic-speaking participants, formal psychometric validation of the instruments in this subgroup within the Turkish context has not been established, and their results should be interpreted with caution. Several potentially important confounders—including medication adherence, socioeconomic status, family functioning, and transplant indication—were not incorporated into the regression models, either due to sample size constraints or the limitations of retrospective clinical records. Finally, although the absence of rejection episodes and low hospitalization rates confirm the medical stability of this cohort, the findings may not extend to higher-risk recipients with active graft complications. Longitudinal studies are needed to clarify the temporal relationships between psychiatric burden and quality of life in this population.

## 5. Conclusions

Given the exploratory, cross-sectional design and relatively small sample size, all findings reported here should be interpreted as hypothesis generating rather than confirmatory. This study revealed the continuous psychiatric challenges faced by PLT recipients, with behavioral, depressive, anxiety, and post-traumatic symptoms associated with worse HRQOL, particularly in the physical, emotional, and social domains. Psychosocial screening accounting for personal and family characteristics appears to be essential in designing and planning post-transplant care. We also believe that utilizing instruments such as the CBCL and the SCARED may enable early detection and thus intervention. By addressing modifiable factors such as parental support and trauma processing, it may be possible to increase resilience and improve HRQOL for these children.

## Figures and Tables

**Table 1:** Demographic and Clinical Characteristics of the Study Population.

Age of Child, Years	14 (11–16)
Sex	
Male	28 (56.0%)
Female	22 (44.0%)
Height, cm	155.5 (135–165)
Weight, kg	45.10 ± 15.94
Body mass index, kg/m^2^	19.38 ± 4.16
Comorbidity	2 (4.0%)
Immigrant	7 (14.0%)
Going to school	36 (72.0%)
Age of mother, years	38.10 ± 5.98
Education status of mother	
Literate	17 (34.0%)
Primary school	17 (34.0%)
Secondary school	7 (14.0%)
High school	5 (10.0%)
Associate degree	3 (6.0%)
Bachelor degree	1 (2.0%)
Age of father, years	42.08 ± 6.03
Education status of father	
Literate	8 (16.0%)
Primary school	13 (26.0%)
Secondary school	17 (34.0%)
High school	8 (16.0%)
Associate degree	1 (2.0%)
Bachelor degree	3 (6.0%)
Chronic disease in family	10 (20.0%)
Age at transplantation, years	5 (2–11)
Time since transplantation, years	7.44 (2.25–11.67)
Type of donor	
Living	44 (88.0%)
Deceased	6 (12.0%)
Immunosuppressive drug	
Tacrolimus	43 (86.0%)
Everolimus	7 (14.0%)
Transplantation complication	0 (0.0%)
Rejection attack, last year	0 (0.0%)
Hospitalization, last year	2 (4.0%)
AST, U/L	32 (25–58)
ALT, U/L	27 (16–71)
Total bilirubin, mg/dL	0.60 (0.42–0.88)
GGT, U/L	27.5 (17–93)

Descriptive statistics are presented using the mean ± standard deviation for normally distributed continuous variables, median (25th percentile–75th percentile) for non-normally distributed continuous variables and frequency (percentage) for categorical variables. Abbreviations: ALT: Alanine aminotransferase, AST: Aspartate aminotransferase, GGT: Gamma-glutamyl transferase.

**Table 2:** Psychological Assessment and Quality of Life Outcomes.

Psychiatric Follow-Up	1 (2.0%)
Psychiatric diagnosis	0 (0.0%)
Type of interview	
Face to face	30 (60.0%)
Online	20 (40.0%)
CBCL Total score	43.5 (36–57)
CDI score	8.14 ± 5.47
Normal (<15)	42 (84.0%)
Mild depression (15–19)	6 (12.0%)
Moderate depression (20–24)	2 (4.0%)
Severe depression (≥25)	0 (0.0%)
SCARED score	
Panic/Somatic	1 (0–2)
≥7	4 (8.0%)
Generalized Anxiety	0 (0–2)
≥9	4 (8.0%)
Separation Anxiety	3 (1–5)
≥5	15 (30.0%)
Social Anxiety	4.40 ± 2.84
≥8	5 (10.0%)
School Avoidance	0 (0–1)
≥3	5 (13.9%)
Total	10 (6–12)
≥25	6 (12.0%)
CRIES-13 score	
Intrusion	1.5 (0–5)
Avoidance	2.5 (0–7)
Arousal	3.5 (1–7)
Total	8 (3–19)
PedsQL score	
Physical Health	71.88 (53.13–93.75)
Emotional Functioning	92.5 (85–100)
Social Functioning	80 (65–100)
School Functioning	63.29 ± 19.25
Psychosocial Health	78.90 ± 13.14
Total	75.71 ± 14.57

Descriptive statistics are presented using the mean ± standard deviation for normally distributed continuous variables, median (25th percentile–75th percentile) for non-normally distributed continuous variables and frequency (percentage) for categorical variables. Abbreviations; CBCL: Child Behavior Checklist, CDI: Children’s Depression Inventory, CRIES-13: Children’s Revised Impact of Events Scale 13-item, SCARED: Screen for Child Anxiety Related Disorders, PedsQL: Pediatric Quality of Life Inventory. The SCARED School Avoidance subscale was administered only to the 36 participants who were attending school at the time of assessment; the percentage for threshold-exceeding scores in this subscale is calculated relative to this subgroup.

**Table 3:** Correlations between PedsQL scores and CBCL, CDI, SCARED, CRIES-13 scores.

		PedsQL					
		Physical Health	Emotional Functioning	Social Functioning	School Functioning	Psychosocial Health	Total
CBCL Total	r	−0.417 ^‡^	−0.778 ^‡^	−0.406 ^‡^	−0.140 ^‡^	−0.608 ^‡^	−0.607 ^‡^
	*p*	**0.003**	**<0.001**	**0.003**	0.422	**<0.001**	**<0.001**
CDI	r	−0.365 ^‡^	−0.688 ^‡^	−0.284 ^‡^	−0.197 ^†^	−0.467 ^†^	−0.446 ^†^
	*p*	**0.009**	**<0.001**	**0.045**	0.257	**0.001**	**0.001**
SCARED Panic/Somatic	r	−0.278 ^‡^	−0.678 ^‡^	−0.177 ^‡^	0.101 ^‡^	−0.390 ^‡^	−0.397 ^‡^
	*p*	0.051	**<0.001**	0.219	0.565	**0.005**	**0.004**
SCARED Generalized Anxiety	r	−0.275 ^‡^	−0.757 ^‡^	−0.211 ^‡^	0.084 ^‡^	−0.539 ^‡^	−0.439 ^‡^
	*p*	0.053	**<0.001**	0.142	0.630	**<0.001**	**0.001**
SCARED Separation Anxiety	r	−0.147 ^‡^	−0.514 ^‡^	−0.213 ^‡^	−0.110 ^‡^	−0.370 ^‡^	−0.318 ^‡^
	*p*	0.310	**<0.001**	0.137	0.529	**0.008**	**0.025**
SCARED Social Anxiety	r	−0.511 ^‡^	−0.516 ^‡^	−0.304 ^‡^	−0.223 ^†^	−0.480 ^†^	−0.582 ^†^
	*p*	**<0.001**	**<0.001**	**0.032**	0.198	**<0.001**	**<0.001**
SCARED School Avoidance	r	−0.373 ^‡^	−0.595 ^‡^	−0.099 ^‡^	−0.157 ^‡^	−0.409 ^‡^	−0.457 ^‡^
	*p*	**0.025**	**<0.001**	0.566	0.367	**0.013**	**0.005**
SCARED Total	r	−0.403 ^‡^	−0.745 ^‡^	−0.312 ^‡^	−0.087 ^‡^	−0.510 ^‡^	−0.557 ^‡^
	*p*	**0.004**	**<0.001**	**0.028**	0.620	**<0.001**	**<0.001**
CRIES−13 Intrusion	r	−0.169 ^‡^	−0.642 ^‡^	−0.320 ^‡^	−0.075 ^‡^	−0.522 ^‡^	−0.400 ^‡^
	*p*	0.239	**<0.001**	**0.023**	0.667	**<0.001**	**0.004**
CRIES-13 Avoidance	r	−0.216 ^‡^	−0.436 ^‡^	−0.376 ^‡^	−0.181 ^‡^	−0.470 ^‡^	−0.396 ^‡^
	*p*	0.132	**<0.001**	**0.007**	0.297	**0.001**	**0.004**
CRIES-13 Arousal	r	−0.434 ^‡^	−0.665 ^‡^	−0.225 ^‡^	−0.165 ^‡^	−0.544 ^‡^	−0.575 ^‡^
	*p*	**0.002**	**<0.001**	0.117	0.342	**<0.001**	**<0.001**
CRIES-13 Total	r	−0.336 ^‡^	−0.676 ^‡^	−0.340 ^‡^	−0.207 ^‡^	−0.605 ^‡^	−0.548 ^‡^
	*p*	**0.017**	**<0.001**	**0.016**	0.234	**<0.001**	**<0.001**

^†^ Pearson correlation coefficient, ^‡^ Spearman correlation coefficient. Statistically significant *p* values are shown in bold. Abbreviations: CBCL: Child Behavior Checklist; CDI: Children’s Depression Inventory; CRIES-13: Children’s Revised Impact of Events Scale-13; PedsQL: Pediatric Quality of Life Inventory; r: Correlation coefficient; SCARED: Screen for Child Anxiety Related Disorders. The choice between Pearson (^†^) and Spearman (^‡^) correlation for each variable pair was determined by the result of normality assessment using histograms and Q-Q plots.

**Table 4:** Associations between PedsQL Total score, linear regression analysis results.

	Univariable			Multivariable ^(a)^		
	Unstandardized Coefficients (95% CI)	Standardized Coefficients	*p*	Unstandardized Coefficients (95% CI)	Standardized Coefficients	*p*
Age of child, years	1.400 (0.084–2.715)	0.295	**0.038**			0.081
Sex, Female	−4.719 (−13.042–3.604)	−0.162	0.260			
Body mass index, kg/m^2^	0.283 (−0.731–1.297)	0.081	0.577			
Comorbidity, Yes	−11.494 (−32.598–9.610)	−0.156	0.279			
Immigrant, Yes	0.670 (−11.395–12.735)	0.016	0.912			
Going to school, Yes	−2.047 (−11.353–7.259)	−0.064	0.660			
Age of mother, years	0.259 (−0.443–0.962)	0.107	0.461			
Education status of mother	0.114 (−3.123–3.352)	0.010	0.944			
Age of father, years	−0.069 (−0.774–0.637)	−0.029	0.846			
Education status of father	−0.150 (−3.419–3.119)	−0.013	0.927			
Chronic disease in family, Yes	−10.134 (−20.180–−0.088)	−0.281	**0.048**			0.072
Age at transplantation, years	−0.197 (−1.060–0.667)	−0.066	0.649			
Time since transplantation, years	0.777 (−0.049–1.603)	0.263	0.065			
Type of donor, Deceased	−10.081 (−22.628–2.467)	−0.227	0.113			
Immunosuppressive drug, Everolimus	−1.406 (−13.466–10.654)	−0.034	0.816			
Hospitalization, last year	−19.421 (−40.031–1.189)	−0.264	0.064			
AST	0.031 (−0.136–0.197)	0.053	0.714			
ALT	0.035 (−0.051–0.122)	0.118	0.414			
Bilirubin	−0.320 (−1.602–0.961)	−0.072	0.618			
GGT	0.007 (−0.046–0.061)	0.040	0.783			
Psychiatric follow-up, Yes	−24.020 (−53.103–5.063)	−0.233	0.103			
Type of interview, Online	7.661 (−0.591–15.914)	0.260	0.068			
CBCL Total score	−0.408 (−0.611–−0.206)	−0.505	**<0.001**			0.279
CDI score	−1.189 (−1.881–−0.497)	−0.446	**0.001**			0.475
SCARED Total score	−0.719 (−1.088–−0.350)	−0.493	**<0.001**	−0.431 (−0.824–−0.038)	−0.295	**0.032**
CRIES−13 Total score	−0.642 (−0.928–−0.356)	−0.546	**<0.001**	−0.471 (−0.787–−0.154)	−0.400	**0.004**
Adjusted R^2^	-			0.337		
Regression model	-			F = 13.436, *p* < 0.001		

(a) Multivariable analysis was performed via stepwise selection method to avoid multicollinearity. Variance inflation factor (VIF) of the variables in the final model is 1.321. Statistically significant *p* values are shown in bold. Abbreviations: ALT: Alanine aminotransferase, AST: Aspartate aminotransferase, CBCL: Child Behavior Checklist, CDI: Children’s Depression Inventory, CI: Confidence interval, CRIES-13: Children’s Revised Impact of Events Scale 13-item, GGT: Gamma-glutamyl transferase, PedsQL: Pediatric Quality of Life Inventory, SCARED: Screen for Child Anxiety Related Disorders, and VIF: Variance inflation factor.

## Data Availability

Data supporting the findings of this study are available from the corresponding author upon reasonable request.

## Abbreviations

CBCL	Child Behavior Checklist
CDI	Children’s Depression Inventory
SCARED	Screen for Child Anxiety Related Disorders
CRIES-13	Children’s Revised Impact of Events Scale-13
PedsQL	Pediatric Quality of Life Inventory
HRQOL	health-related quality of life
PLT	Pediatric liver transplantation
DSM	Diagnostic and Statistical Manual of Mental Disorders
AST	aspartate aminotransferase
ALT	alanine aminotransferase
GGT	gamma-glutamyl transferase
